# Integrated analysis approaches to decode functional consequences of molecular quantitative trait loci for complex traits

**DOI:** 10.1002/alz.092046

**Published:** 2025-01-03

**Authors:** Daniel Nachun

**Affiliations:** ^1^ Stanford University, Palo Alto, CA USA

## Abstract

**Background:**

Genome‐wide association studies (GWAS) have identified thousands of genomic regions associated with complex diseases but understanding the underlying causal mechanisms remains a significant challenge. The FunGen‐xQTL project has addressed this by generating and harmonizing molecular quantitative trait loci (xQTL) across multiple layers of molecular traits in human brains, cerebrospinal fluid, and blood‐derived cells relevant to neurodegenerative disorders. Existing approaches for integrating xQTL data with GWAS have typically focused on individual molecular traits in individual QTL layers. There is a scarcity of systematic, rigorous computational methods that effectively utilize the diverse data from the FunGen‐xQTL project to understand the convergence of complex disease pathogenesis across various ‘omics levels.

**Methods:**

FunGen‐xQTL has evaluated current best practices for xQTL integration with GWAS, creating standardized analysis protocols and bioinformatics workflows for a chosen set of methods. We have developed new computational tools for many tasks: Functional fine mapping for methylation, histone acetylation and chromatin accessibility QTLs (*fSuSiE*)

• Multi‐omic fine mapping for joint analysis of different ‘omics layers (*mvSuSiE*)

• Pairwise enrichment, colocalization, TWAS and Mendelian randomization (*pecotmr*)

• Multi‐omic colocalization with gradient boosting (*colocBoost*)

• Multi‐omic and multi‐trait TWAS and Mendelian randomization (*mr.mash*, *CTWAS*)

• Multi‐omic discovery of transQTL effects on gene networks (*transCCA*)

• Improved predictive models for identifying rare variants that impact function genomics (*Watershed2*)

• Improved quantification of splicing for splicing QTL mapping (*leafcutter2*).

**Results:**

In both simulated and real data, we have found that our novel methods outperform existing methods for discovering associations between xQTL and GWAS when applied to different xQTL types. We have prepared precompiled packages for the *conda* family of package managers which are bundled together into containers for each workflow that can be deployed on cloud providers or local computing clusters. We have designed different schemes for xQTL integration, including disease‐centric, gene‐centric, and event‐centric analysis, to characterize the functional landscape of xQTLs around AD loci.

**Conclusions:**

Our novel tools have improved our ability to identify shared genetic etiology between multiple related molecular traits, metabolic pathways, and Alzheimer’s disease (AD) that are highlighted by the other talks in this session.

